# Antidepressant Fluoxetine Modulates the In Vitro Inhibitory Activity of Buffalo Brain Cystatin: A Thermodynamic Study Using UV and Fluorescence Techniques

**DOI:** 10.1155/2014/319397

**Published:** 2014-07-24

**Authors:** Fakhra Amin, Bilqees Bano

**Affiliations:** Department of Biochemistry, Faculty of Life Sciences Aligarh Muslim University, Aligarh 202002, India

## Abstract

Cystatins constitute a superfamily of homologous proteins. The major role of cystatins is to regulate the unwanted proteolysis and to protect the organism against endogenous proteases released from lysosomes, invading microorganisms and parasites that use cysteine proteases to enter the body. Imbalance in regulation of proteolytic activity may lead to a wide range of human diseases. An enormous progress has been made in understanding of protein degradation process under normal and pathological conditions; infact proteases are now clearly viewed as important drug targets. Fluoxetine a selective serotonin reuptake inhibitor (SSRI) is an antidepressant. It is used to treat major depressive disorders. In the present study binding of fluoxetine to cystatin was studied by UV and fluorescence quenching technique. Intrinsic fluorescence of fluoxetine complexed with purified buffalo brain cystatin (BC) was measured by selectively exciting the tryptophan residues. Gradual quenching was observed on complex formation. When cystatin was added to fluoxetine solutions at a molar ratio of 1 : 0.5, it not only quenched more than half of its fluorescence but also reduced the activity of cystatin. Stern-Volmer plots obtained from experiments carried out at 25^°^C showed the quenching of fluorescence to be a collisional phenomenon. Our results suggest the prime binding site for fluoxetine on BC to be at or near tryptophan residues. Fluoxetine quenched the fluorescence by a static process, which specifically indicates the formation of a complex.

## 1. Introduction

The most studied inhibitors of the papain family are the cystatins. They are present in mammals, birds, insects, plants, and protozoa. They function both intracellularly and extracellularly. Cystatins are competitive, reversible, and tight binding protein inhibitors which display structural and functional similarities. They serve a protective function by regulating the activities of endogenous proteinases, which if not regulated may cause uncontrolled proteolysis and damage to cells and tissues. On the basis of homology, inhibition of target enzymes and presence or absence of disulphide bonds, cystatin superfamily has been divided into three families. Family I also called as stefins include members of low molecular weight proteins (approximately 11 KDa), which lack disulphide bonds and carbohydrate content. This family includes cystatin A, cystatin B, stefin C, and stefin D. Family II known as cystatin family is represented by the inhibitors of a bit larger molecular weight proteins (approximately 13 KDa) compared to stefins and possesses disulphide bonds towards carboxyl terminal. This family comprises cystatins C, D, S, SN, E, F, and M. Family III or kininogens are higher molecular weight inhibitors containing both disulphide linkage and carbohydrate content. They are found only in blood plasma. There are three distinct types of kininogens designated as high molecular weight kininogen HK (MW120 KDa), low molecular weight kininogens LK (MW 50–70 KDa), and T-kininogen found only in rat plasma [1].

Among the bovines there are several species including cow and buffalo and their cystatins were isolated from various organs, mostly from cow and characterized including stefins A, B, and C [2]. Complete amino acid sequence of stefin A, stefin B, and stefin C has been determined which were isolated from bovine thymus [2–4]. According to the nomenclature, stefin C is a member of the stefin family (family I) and should not be confused with cystatin C, a member of the cystatin family (family 11).

Many endogenous compounds that exist in human bodies can bind with drugs to form stable complexes, which interfere with the functions of regulating proteins directly or indirectly [5, 6]. In addition, the effectiveness of drugs depends on their binding ability. It has been shown that the distribution, free concentration, and the metabolism of various drugs may be strongly affected by drug proteins interaction in the blood stream [7–10]. Drug interactions in most cases significantly affect the apparent distribution volume of the drugs and also affect the elimination rate and determine the therapeutic affectivity of drugs [11].

Therefore, study of the interaction between proteins and drug molecules helps to provide basic information on the pharmacological action, biotransformation, and biodistribution of drugs [12]. Studying the interaction of drugs with proteins by the fluorescence techniques is commonly used because of its high sensitivity, rapidity, and ease of interpretation.

Several reports have been published using this technique for the interaction of proteins with drugs [13–15].

An antidepressant is a psychiatric medication used to alleviate mood disorders and major depression. Drugs including the monoamine oxidase inhibitors (MAOIs), tricyclic antidepressants (TCAs), selective serotonin reuptake inhibitors (SSRIs), and serotonin-norepinephrine reuptake inhibitors (SNRIs) are most commonly associated with the term.

**Figure 1:**
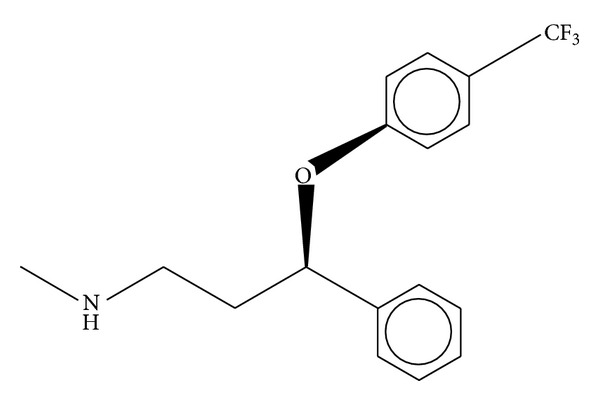
Structure of fluoxetine [13].

Fluoxetine (trade name Prozac) is an antidepressant of the (SSRI) class (selective serotonin reuptake inhibitor) (Figure 1). Fluoxetine is approved for the treatment of major depressions, anorexia nervosa, and panic disorder [16]. The bioavailability of fluoxetine is relatively high (72%), and peak plasma concentrations are reached in 6 to 8 hours. It significantly binds to plasma proteins, mostly albumin. Fluoxetine is metabolized in the liver by isoenzymes of the cytochrome P450 system; only one metabolite of fluoxetine, norfluoxetine (demethylated fluoxetine), is biologically active. The extremely slow elimination of fluoxetine and its active metabolite norfluoxetine from the body distinguishes it from other antidepressants [17–19]. Understanding the downstream effects and complexity of protease inhibitors and their study with antidepressant is therefore a challenging but crucial part of protease function regulation because unexpected drug interactions with regulatory proteins in the cascade can have devastating effects on the safety profile of a drug.

## 2. Material and Methods

### 2.1. Materials

**Figure 2:**
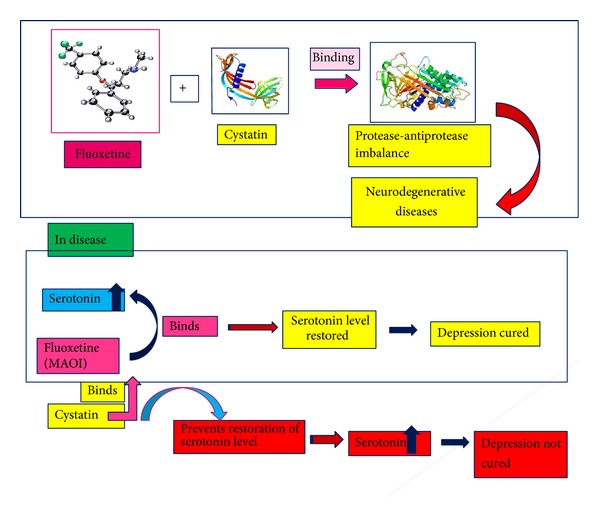
Proposed work.

Papain 99% purity was obtained from Sigma Chemical Company (St. Louis, USA). Fluoxetine (an antidepressant drug) was purchased from Ranbaxy (India). The solutions were prepared in 50 mM phosphate buffer of pH 7.4. Salts of different metals, phosphate were purchased from Merck (India). The protein concentration was determined spectrophotometrically. All other materials were of analytical reagent grade and double distilled water was used throughout (Figure 2).

### 2.2. Apparatus

The absorbance spectra were recorded on a double beam Shimadzu UV-Vis spectrophotometer UV-1700 using a cuvette of 1 cm path length. Fluorescence measurements were performed on a spectrofluorometer Model RF-5301PC (Shimadzu, Japan) equipped with a 150 W Xenon lamp and a slit width of 10 nm. A 1.00 cm quartz cell was used for measurements.

### 2.3. Purification of Brain Cystatin

Fresh brain tissue (150 grams) was homogenized in 50 mM sodium phosphate buffer of pH 7.5 (30 mL) containing 1% NaCl, 3 mM EDTA, and 2% n-butanol. After centrifugation at 11000 rpm for 15 minutes at 4°C residue was discarded and the supernatant was further processed. The procedure involved a combination of alkaline treatment (pH 11.0), ammonium sulphate fractionation, and gel filtration chromatography. Buffalo brain was homogenized and fractionated with ammonium sulfate between 40–60%; it was then dialyzed against 50 mM sodium phosphate buffer pH 7.4 containing 0.1 M NaCl. Elution profile showed two protein peaks one major and one minor named as peak-I and peak-II. Peak-I corresponding to high molecular weight. Cystatin had significant inhibitory activity and protein content; however peak-II with insignificant proteins concentration and low inhibitory activity was not taken into consideration for further studies. Peak-I renamed as BC was then purified with fold purification of 384.72 and yield of 64.13%. Papain inhibitory fractions of peak-I were pooled, concentrated, and checked for purity. Five milliliter fractions were collected and assayed for protein by the method of Lowry et al. (1951) and inhibitory activity against papain was determined by the method of Kunitz (1947). Homogeneity of the preparation was investigated by 7.5% PAGE [20].

## 3. Spectroscopic Studies

### 3.1. Fluorescence Spectra of Brain Cystatin with Fluoxetine

Brain cystatin (BC) (1 *μ*M) was incubated for 30 min with increasing concentration of drug in 0.05 M sodium phosphate buffer pH 7.5 in a final reaction volume of 1 mL at room temperature. Drug solutions were prepared in the same buffer. Fluorescence measurements were carried out on a Shimadzu Spectrofluorometer model RF-5301PC (Shimadzu, Japan) equipped with a 150 W Xenon lamp and a slit width of 10 nm at 298 K. The fluorescence was recorded in wavelength region 300–400 nm after exciting the protein at 280 nm. The slits were set at 10 nm for excitation and emission. The path length of the sample was 1 cm. The data was analyzed by Stern-Volmer equation.

### 3.2. Stern-Volmer Constant

The fluorescence quenching was analyzed by the Stern-Volmer equation

(1) $$\begin{array}{c} \frac{F_{0}}{F}=1+K_{\text{sv}}\left[Q\right], \end{array}$$

where *F*_0_ and *F* are the fluorescence intensities in the absence and presence of quencher, respectively, *K*_sv_ is the Stern-Volmer quenching constant, and [*Q*] is the concentration of the quencher.

### 3.3. Determination of Binding Constant [*K*] and Number of Binding Sites (*n*)

When small molecules bind independently to set of equivalent sites on macromolecules, the equilibrium between free and bound molecules is given by the following equation [21, 22]:

(2) $$\begin{array}{c} \text{Log}\frac{\left(F_{0}-F\right)}{F}=\text{Log}K+n\text{Log}\left[Q\right], \end{array}$$

where *K* and *n* are the binding constant and number of binding sites, respectively; thus a plot of Log⁡(*F*_0_ − *F*)/*F* versus [*Q*] can be used to determine *K* as well as *n*.

### 3.4. Calculation of the Free Enthalpy Δ*G*

The determination of the change of free enthalpy based on the Van't Hoff equation:

(3) $$\begin{array}{c} \Delta G=-RT\ln K\;\left[\frac{\text{J}}{\text{mol}}\right],\;\Delta G=-RT\ln K\;\;{\text{Jmol}}^{-1}, \end{array}$$

where *R* is the gas constant (8.314(J/mol × K))8.314 Jmol × K, *T* is the temperature [K], and *K* is the equilibrium constant.

### 3.5. UV Spectra of Cystatin in the Presence of Antidepressant

The UV measurement of brain cystatin in the presence and absence of antidepressants was made in the range of 200–300 nm and the inhibitor (cystatin) concentration was fixed at 1 *μ*M while the drug concentration was varied for different drugs to different extent. Absorption spectra were recorded on a double beam Shimadzu UV-vis spectrophotometer UV-1700 using a cuvette of 1 cm path length.

### 3.6. Activity Measurement of Brain Cystatin in the Presence of Drug Fluoxetine

The inhibitory activity of the purified inhibitor (BC) under native conditions was assessed by its ability to inhibit caseinolytic activity of papain by the method of Kunitz [23]. The inhibitor (1 *μ*M) was incubated with increasing concentrations of drugs at 25°C for 30 min before the activity was measured. Activity of untreated BC was taken as 100%.

## 4. Results

### 4.1. Interaction of Fluoxetine with Brain Cystatin

#### 4.1.1. Fluorescence Spectra of Fluoxetine with Brain Cystatin

**Figure 3:**
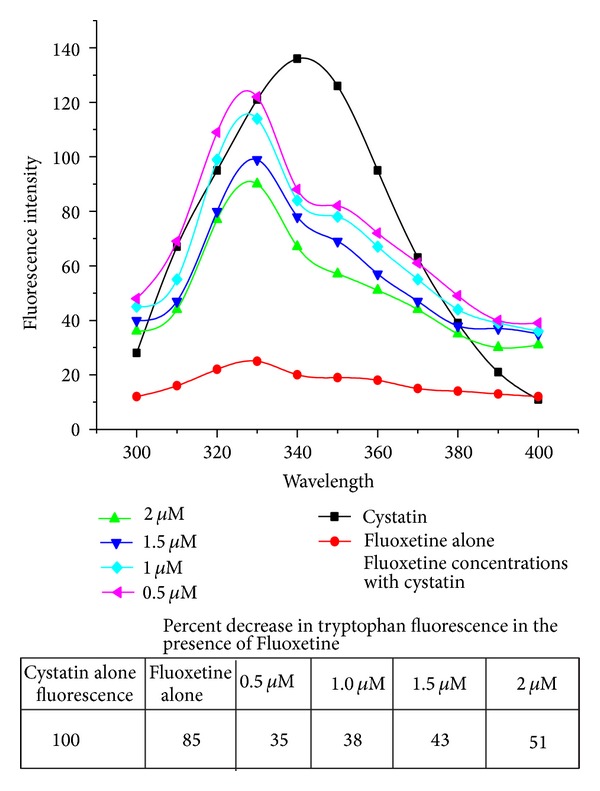
Intrinsic fluorescence study of cystatin in the presence and absence of fluoxetine. BC (1 *μ*M) was incubated with various concentrations of fluoxetine varying from 0.5 *μ*M to 2 *μ*M for 30 min. The fluorescence was recorded in the wavelength region 300–400 nm, exciting the protein solution at 280 nm. The slits were set at 10 nm for excitation and emission. The path length of the sample was 1 cm.

In this study fluorescence spectra of Cystatin (1 *μ*M) in the presence of different concentrations of fluoxetine were recorded in the range of 300–400 nm upon excitation at 280 nm. The drug caused quenching of the intrinsic fluorescence of cystatin (Figure 3) with 10 nm of blue shift in wavelength. As the concentration of fluoxetine increases, fluorescence intensity decreases; maximum decrease in fluorescence intensity occurred at 2 *μ*M of drug concentration leading to quenching up to 51%. These results indicated that there were interactions between fluoxetine and cystatin (BC); moreover the binding reactions resulted in nonfluorescent complex.

#### 4.1.2. The Fluorescence Quenching Data Was Analysed by the Stern-Volmer Equation as Described Earlier for Fluoxetine

*K*_sv_ the Stern-Volmer quenching constant value indicates the affinity of binding obtained at 298 K where as in Table 1.

### 4.2. Determination of Binding Constant (*K*) and Number of Binding Sites (*n*)

These values were calculated as described earlier in Methods section. The value of binding constant *K* was found to be 5.03 × 10^6^ mol^−1^ and the number of binding sites was equal to 1 for fluoxetine (Table 1).

### 4.3. Δ*G* ^0^ of Interaction between Fluoxetine and Cystatin

Free energy change (Δ*G* ^0^) of the interactions was calculated as described in Methods section. The value was found to be −38.2 KJ/mol showing the reaction to be spontaneous (Table 1).

### 4.4. UV-Vis Absorption Studies of Fluoxetine Cystatin Complex

**Figure 4:**
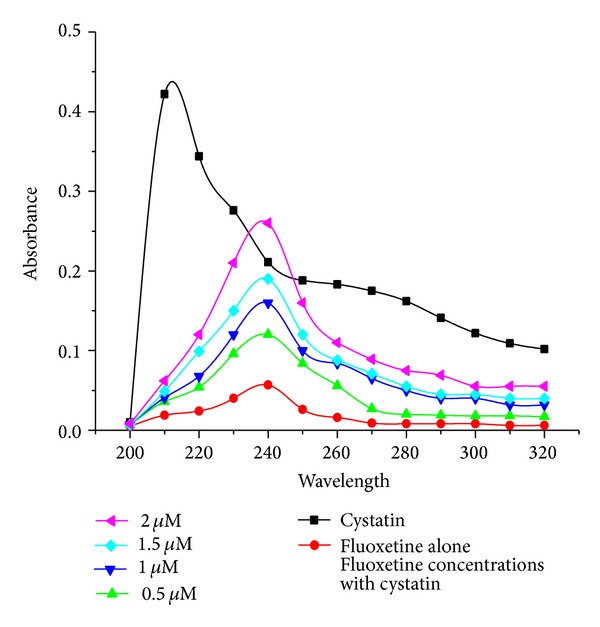
UV-vis spectroscopy of cystatin in the presence and absence of fluoxetine. BC concentrations were fixed at 1 *μ*M while the concentration of fluoxetine was varied from 0.5 *μ*M to 2 *μ*M. Absorption spectra of native BC and in the presence and absence of fluoxetine were recorded in the range of 200–300 nm, cuvette of 1 cm path length for 30 min in the final reaction volume of 1 mL in 0.05 M sodium phosphate buffer pH 7.5.

The interaction between fluoxetine and cystatin was also studied from UV-vis absorption spectral data. Cystatin concentrations were fixed at 1 *μ*M while the fluoxetine concentration was varied from 0.5 *μ*M to 2 *μ*M. Absorption spectra of cystatin in the presence of fluoxetine were recorded in the range of 200–300 nm. The UV absorption intensity of cystatin increased with the variation of fluoxetine concentration. UV absorbance spectra of cystatin, fluoxetine, and their complexes are shown in (Figure 4). Cystatin showed peak in the region 200–210 nm, while on complexation with fluoxetine profound changes were introduced and there was peak shift of 30 nm (red shift) with enhanced absorbance as compared to fluoxetine. The UV-vis absorption spectra were computed at all the Ffluoxetine concentrations. However, little change was noted between 0.5 and 2 *μ*M fluoxetine. The spectra obtained for cystatin interaction with 2 *μ*M, fluoxetine showed peaks at 240 nm, the gross conformation of BC at all concentrations of fluoxetine was not effected significantly [24, 25].

### 4.5. Inhibitory Activity of Brain Cystatin in the Presence of Fluoxetine

The results obtained indicate that inactivation of brain cystatin by fluoxetine is concentration dependent. 1 *μ*M cystatin was incubated with increasing concentrations of fluoxetine (0.5–2 *μ*M) in 50 mM sodium phosphate buffer pH 7.5 at room temperature for 30 min; its inhibitory activity was determined by caseinolytic assay of papain [23]. The activity of native cystatin was taken as 100%. On interaction with 0.5 *μ*M fluoxetine, 48% loss of cystatin activity was noticed (Table 2). However at 1 *μ*M drug concentration 52% of inhibitor activity was compromised, a drastic decline (90%) was noticed at 2 *μ*M Fluoxetine drug concentration, and the inhibitor retained only 10% of its original papain inhibition potential.

## 5. Discussion

Cystatins are the inhibitors of cysteine proteinases, most of which form equimolar complexes with their target enzymes. Cysteine proteinase inhibitors of cystatin superfamily are present in a variety of tissues and body fluids of human beings and animals to regulate the activities of cysteine proteinases.

In addition to natural inhibitors some synthetic inhibitors like endopin 2C modified as serpinendopin 2C show selective inhibition of cathepsin L compared to papain or elastase [26]; other inhibitors like peptidyl-diazomethyl ketones are also useful irreversible inhibitors for inactivating cysteinyl proteinases in vitro and in vivo more effectively against cathepsin L than cathepsin S [27]. These peptidyl-diazomethyl ketone inhibitors have been found to be very fast and irreversible inhibitors of cysteine proteases [28]. Both peptidyl-diazoethyl and chloroethyl ketones were much less potent inhibitors for cathepsins B of the papain family of cysteine proteinases [29].

E-64 isolated from cultures of* Aspergillus japonicus* is a very strong and irreversible inhibitor of cysteine proteases [30, 31].

Cystatins the crucial inhibitors for proper brain functioning have been reported from several mammalian sources and an imbalance of proteinases (cathepsins) with their endogenous inhibitor cystatins is closely associated with senile plaque, cerebrovascular amyloid deposits, and neurofibrillary tangles in Alzheimer's disease. It has also been reported that cystatin C is present in high concentration in CNS and is suggested to play an important role in diseases of the brain [32]. A proteinase inhibitor is of physiological importance because inhibition is achieved at physiological concentration of the inhibitor in a sufficiently short time with negligible dissociation of the complex. Endogenous thiol proteinase inhibitors the cystatins constitute a powerful regulatory system for overall cellular activity of cysteine proteinases [33].

Moreover they are associated with several neurodegenerative diseases and pathological conditions including rheumatoid arthritis [34], osteoporosis [35], renal failure, and cardiovascular and cancer diseases [36, 37].

Fluoxetine is a selective serotonin uptake inhibitor which is clinically useful in treating depression and may also be useful for management of a variety of other psychiatric and metabolic derangements. Fluoxetine antagonizes the neurotoxic effects of p-chloroamphetamine, a compound that depletes serotonin [38, 39]. Fluoxetine is effective in the treatment of depression [40] and obesity [41].

Fluorescence technique has been widely used for drug-protein interaction studies [42, 43]. In this study the addition of increasing concentrations of fluoxetine caused a progressive reduction of the fluorescence intensity of the complex (Figure 1) with 10 nm of blue shift in the wavelength *λ*_max⁡_ (emission maximum). Thus, the fluorescence was strongly quenched, whereas *λ*_max⁡_ was decreased from 340 to 330 nm by the addition of 2 *μ*M of Fluoxetine, the shift in fluorescence intensity can reasonably be attributed to the increased hydrophobicity (or a decreased polarity) of the region surrounding the tryptophan site [13]. Similar spectral features were observed for the interaction of compound [Zn(L_2_)(phen)] with BSA.

Studies on the binding mechanism between protein and small molecules provide useful information. For example, a detailed characterization of drug-protein binding properties was essential for understanding the function of drugs and hence interest in drug-protein interaction has attracted much attention and the development of drugs based on inhibition of cystatins has advanced into clinical testing with targeting compounds [1].

The static type of quenching is indicative of a complex formation between the protein and the drug molecule.

Stern-Volmer equation is used to study the interaction of cystatin with fluoxetine. The interaction forces between proteins and ligands may comprise hydrophobic, hydrogen bonds, van der Waals, and electrostatic interactions [44]. The free energy change (Δ*G* ^0^) is estimated for the interaction of fluoxetine with cystatin which is shown in (Table 1). The negative values of the free energy (Δ*G* ^0^) support the assertion that the binding process is spontaneous.

The UV-vis absorption difference spectra were computed at all the drug concentrations. However, profound changes were noted only for those obtained at 0.5–2 *μ*M fluoxetine (Figure 4). The spectra obtained for fluoxetine which interacted with 1 *μ*M cystatin show peaks at 240 nm with shift of 30 nm. The data was used for calculating the Stern-Volmer constant.

When 1 *μ*M Cystatin was incubated with increasing concentrations of the fluoxetine (0.5–2 *μ*M), its inhibitory activity decreased in concentration dependent manner. On interaction with 0.5 *μ*M fluoxetine 48% loss of cystatin activity was noticed (Table 2). At 1 *μ*M drug concentration 52% of inhibitor's activity was compromised. A drastic decline (90%) was noticed at 2 *μ*M drug concentration.

This could be explained by the fact that fluoxetine is a competitive inhibitor of serotonin uptake which may interact with the same portion of the carrier protein responsible for the transport of serotonin; the structural features responsible for substrate-carrier protein recognition may be different from those responsible for inhibitor-carrier protein recognition [45]. From this study, accurate measurements of fluoxetine binding properties are expected to open the door to new avenues in the screening and design of appropriate antidepressant drugs that may be of importance in modern medical research. Additional studies are required to determine whether the structural overlap between fluoxetine and serotonin is biochemically and pharmacologically meaningful.

## Figures and Tables

**Table 1:** Different parameters of the drug fluoxetine obtained by Stern-Volmer equation for interaction with cystatin.

Drug parameter	*K*_sv_ (Stern-Volmer constant) mol^−1^	*K* (binding constant) mol^−1^	*n* (number of binding sites)	Δ*G* ^0^ (free energy change) KJ/mol
Fluoxetine	0.5 × 10^6^	5.0317 × 10^6^	0.79	−38.232

**Table 2:** Inhibitory activity of brain cystatin in the presence of fluoxetine. The table shows changes in the inhibitory activity of brain cystatin after incubation for 30 min with increasing concentration of fluoxetine.  BC  (1 *μ*M) treated with varying concentrations of fluoxetine (0.5 *μ*M–2 *μ*M) for 30 min in the final reaction volume of 1 mL in 0.05 M sodium phosphate buffer pH 7.5.

S. number	Fluoxetine concentration	% Inhibitory activity remaining
1	Cystatin alone	100
2	Cystatin + 0.5 *μ*M fluoxetine	52 ± 0.394
3	Cystatin + 1 *μ*M fluoxetine	48 ± 0.770
4	Cystatin + 1.5 *μ*M fluoxetine	25 ± 0.911
5	Cystatin + 2 *μ*M fluoxetine	10 ± 0.518

All data are expressed as mean ± S.E  for four different sets of experiments; statistical significance was conducted employing oneway ANOVA.   A probability level of 0.05 was selected showing results are significant.

## Abbreviations

BC: Brain cystatin
MAOI: Monoamine oxidase inhibitor; antidepressant
MAO: Monoamine amine oxidase.

## References

[1] Turk V, Turk B (2008). Lysosomal cysteine proteases and their protein inhibitors: recent developments. *Acta Chimica Slovenica*.

[2] Turk B, Ritonja A, Björk I, Stoka V, Dolenc I, Turk V (1995). Identification of bovine stefin A: a novel protein inhibitor of cysteine proteinases. *FEBS Letters*.

[3] Krizaj I, Turk B, Turk V (1992). The complete primary structure of bovine stefin B. *FEBS Letters*.

[4] Turk B, Krizaj I, Kralj B (1993). Bovine stefin C, a new member of the stefin family. *The Journal of Biological Chemistry*.

[5] Silva D, Cortez CM, Louro SR (2004). Chlorpromazine interactions to sera albumins. A study by the quenching of fluorescence. *Spectrochimica Acta A: Molecular and Biomolecular Spectroscopy*.

[6] Huang B, Zou GL, Yang TM (2002). Studies on the interaction between adriamycin and bovine serum albumin. *Acta Chimica Sinica*.

[7] Kamat BP, Seetharamappa J (2004). In vitro study on the interaction of mechanism of tricyclic compounds with bovine serum albumin. *Journal of Pharmaceutical and Biomedical Analysis*.

[8] Seedher N (2000). In vitro study of the mechanism of interaction of trifluoperazine dihydrochloride with bovine serum albumin. *Indian Journal of Pharmaceutical Sciences*.

[9] Seedher N, Bhatia S (2009). Complexation of cox-2 inhibitors with bovine serum albumin: interaction mechanism. *Pharmaceutical Development and Technology*.

[10] Channu BC, Kalpana HN, Eregowda GB, Dass C, Houghton PJ, Thimmaiah KN (1999). Interaction of substituted phenoxazine chemosensitizers with bovine serum albumin. *Journal of Pharmaceutical and Biomedical Analysis*.

[11] Khan SN, Islam B, Rajeswari MR, Usmani H, Khan AU (2008). Interaction of anesthetic supplement thiopental with human serum albumin. *Acta Biochimica Polonica*.

[12] Kragh-Hansen U (1981). Molecular aspects of ligand binding to serum albumin.. *Pharmacological Reviews*.

[13] Tian JN, Liu JQ, Zhang JY, Hu ZD, Chen XG (2003). Fluorescence studies on the interactions of barbaloin with bovine serum albumin. *Chemical and Pharmaceutical Bulletin*.

[14] Sereikaite J, Bumelis VA (2006). Congo red interaction with *α*-proteins. *Acta Biochimica Polonica*.

[15] Khan SN, Islam B, Khan AU (2007). Probing midazolam interaction with human serum albumin and its effect on structural state of protein. *International Journal of Integrative Biology*.

[16] Prozac Pharmacology, Pharmacokinetics, Studies, Metabolism. http://www.rxlist.com/cgi/generic/fluoxetine_cp.htm.

[17] Leo RJ (1996). Movement disorders associated with the serotonin selective reuptake inhibitors. *Journal of Clinical Psychiatry*.

[18] Gerber PE, Lynd LD (1998). Selective serotonin-reuptake inhibitor-induced movement disorders. *The Annals of Pharmacotherapy*.

[19] Caley CF (1997). Extrapyramidal reactions and the selective serotonin-reuptake inhibitors. *Annals of Pharmacotherapy*.

[20] Amin F, Khan A, Rizvi JS, Bano B (2011). Purification and characterization of buffalo brain cystatin. *Protein and Peptide Letters*.

[21] Feng X, Lin Z, Yang L, Wang C, Bai C (1998). Investigation of the interaction between acridine orange and bovine serum albumin. *Talanta*.

[22] Gao H, Lei L, Liu J, Kong Q, Chen X, Hu Z (2004). The study on the interaction between human serum albumin and a new reagent with antitumour activity by spectrophotometric methods. *Journal of Photochemistry and Photobiology A*.

[23] Kunitz M (1947). Crystalline soybean trypsin inhibitor II. General properties. *The Journal of General Physiology*.

[24] Cui FL, Fan J, Li JP, Hu ZD (2004). Interactions between 1-benzoyl-4-*p*-chlorophenyl thiosemicarbazide and serum albumin: investigation by fluorescence spectroscopy. *Bioorganic & Medicinal Chemistry*.

[25] Hu Y-J, Liu Y, Wang J-B, Xiao X-H, Qu S-S (2004). Study of the interaction between monoammonium glycyrrhizinate and bovine serum albumin. *Journal of Pharmaceutical and Biomedical Analysis*.

[26] Hwang S, Stoka V, Turk V, Hook VYH (2005). The novel bovine serpin endopin 2C demonstrates selective inhibition of the cysteine protease cathepsin L compared to the serine protease elastase, in cross-class inhibition. *Biochemistry*.

[27] Shaw E, Mohanty S, Colic A, Stoka V, Turk V (1993). The affinity-labelling of cathepsin S with peptidyl diazomethyl ketones: comparison with the inhibition of cathepsin L and calpain. *FEBS Letters*.

[28] Leary R, Larsen D, Watanabe H, Shaw E (1977). Diazomethyl ketone substrate derivatives as active-site-directed inhibitors of thiol proteases. Papain. *Biochemistry*.

[29] Wikstrom P, Kirschke H, Stone S, Shaw E (1989). The properties of peptidyl diazoethanes and chloroethanes as protease inactivators. *Archives of Biochemistry and Biophysics*.

[30] Barrett AJ, Kembhavi A, Brown MA (1982). L-trans-epoxysuccinyl-leucylamido(4-guanidino)butane (E-64) and its analogues as inhibitors of cysteine proteinases including cathepsins B, H and L. *Biochemical Journal*.

[31] Matsumoto K, Mizoue K, Kitamura K, Tse WC, Huber CP, Ishida T (1999). Structural basis of inhibition of cysteine proteases by E-64 and its derivatives. *Peptide Science*.

[32] Bernstein HG, Kirschke H, Wiederanders B, Pollak KH, Zipress A, Rinne A (1996). The possible place of cathepsins and cystatins in the puzzle of Alzheimer disease. *Molecular and Chemical Neuropathology*.

[33] Sotiropoulou G, Anisowicz A, Sager R (1997). Identification, cloning, and characterization of cystatin M, a novel cysteine proteinase inhibitor, down-regulated in breast cancer. *The Journal of Biological Chemistry*.

[34] Trabandt A, Gay RE, Fassbender H, Gay S (1991). Cathepsin B in synovial cells at the site of joint destruction in rheumatoid arthritis. *Arthritis and Rheumatism*.

[35] Delaisse J-M, Ledent P, Vaes G (1991). Collagenolytic cysteine proteinases of bone tissue: cathepsin B, (pro) cathepsin L and a cathepsin L-like 70 kDa proteinase. *Biochemical Journal*.

[36] Kabanda A, Goffin E, Bernard A, Lauwerys R, van Ypersele de Strihou C (1995). Factors influencing serum levels and peritoneal clearances of low molecular weight proteins in continuous ambulatory peritoneal dialysis. *Kidney International*.

[37] Servais A, Giral P, Bernard M, Bruckert E, Deray G, Isnard Bagnis C (2008). Is serum cystatin-C a reliable marker for metabolic syndrome?. *American Journal of Medicine*.

[38] Wong DT, Bymaster FP, Horng JS, Molloy BB (1975). A new selective inhibitor for uptake of serotonin into synaptosomes of rat brain: 3-(p-trifluoromethylphenoxy). N-methyl-3-phenylpropylamine. *Journal of Pharmacology and Experimental Therapeutics*.

[39] Wong DT, Bymaster FP (1976). The comparison of fluoxetine and nisoxetine with tricyclic antidepressants in blocking the neurotoxicity of p chloroamphetamine and 6 hydroxydopamine in the rat brain. *Research Communications in Chemical Pathology and Pharmacology*.

[40] Stark P, Hardison CD (1985). A review of multicenter controlled studies of fluoxetine vs. imipramine and placebo in outpatients with major depressive disorder. *Journal of Clinical Psychiatry*.

[41] Carruba MO, Ricciardi S, Spano P, Mantegazza P (1985). Dopaminergic and serotoninergic anorectics differentially antagonize insulin- and 2-DG-induced hyperphagia. *Life Sciences*.

[42] Ahmad B, Khan MKA, Haq SK, Khan RH (2004). Intermediate formation at lower urea concentration in 'B' isomer of human serum albumin: a case study using domain specific ligands. *Biochemical and Biophysical Research Communications*.

[43] Yang Y, Hu Q, Fan Y, Shen H (2008). Study on the binding of luteolin to bovine serum albumin. *Spectrochimica Acta A: Molecular and Biomolecular Spectroscopy*.

[44] Timaseff SN, Peters H (1972). Thermodynamics of protein interactions. *Proteins of Biological Fluids*.

[45] (2005). *Clarke's Analysis of Drugs and Poisons*.

